# Subthreshold Micropulse Treatment Laser versus Half-Dose Photo Dynamic Therapy for the Chronic Central Serous Chorioretinopathy with Parafoveal or Subfoveal Leakage STML versus PDT for Treatment of Chronic CSC

**DOI:** 10.1155/2022/3627903

**Published:** 2022-06-30

**Authors:** Qingshan Chen, Xia Zhao, Qiuju Yin, Zhi Li, Zimei Zhao, Jiafeng Ning

**Affiliations:** ^1^Department of Ophthalmology, Shenzhen Eye Disease Prevention & Treatment Institute, Shenzhen Eye Hospital, Jinan University, Shenzhen 518001, China; ^2^Shenzhen Key Laboratory of Ophthalmology, Shenzhen 518001, China; ^3^Jinzhong Aier Eye Hospital, Shanxi 030600, China

## Abstract

**Purpose:**

The study aimed to compare the efficacy and safety outcomes of a subthreshold micropulse treatment laser (SMTL) versus half-dose photodynamic therapy (PDT) for treatment of chronic central serous chorioretinopathy (cCSC) with parafoveal or subfoveal leakage to persistent subretinal fluid.

**Methods:**

One hundred and forty-eight patients (148 eyes) with persistent cCSC were enrolled in this retrospective study and treated with SMTL or half-dose PDT. All patients were diagnosed according to clinical characteristics and findings on multimodal imaging. The medical records including patients with a minimum follow-up period of 3 months were reviewed. The patients were divided into two groups according to the application of the SMTL or PDT. The group of the SMTL or PDT was also divided into two subgroups according to parafoveal or subfoveal leakage. The primary outcomes included best-corrected visual acuity (BCVA) and central retinal thickness (CRT) before and 1, 2, and 3 months after treatment. The repeat treatment and resolution of subretinal fluid were also collected.

**Results:**

Seventy-nine patients (79 eyes) treated with half-dose PDT and 69 Patients (69 eyes) treated with the STML were included. The mean age was 44.20 ± 7.42 years and 80% were male. No significant difference in age, gender, baseline logMAR BCVA or CRT between the half-dose PDT group and the SMTL group (*P*=0.201; 0.051) can be defined. The BCVA of the SMTL group improved at 1, 2, and 3 months after treatment, while that of the half-dose PDT group improved like the SMTL group. There were no significant differences between the SMTL and the PDT group at 1, 2, and 3 months (*P*=0.723; 0.139; 0.896). The CRT for the SMTL group decreased at 1, 2, and 3 months after treatment, while that of the half-dose PDT group changed like the SMTL group. However, there were significant differences between the SMTL group and the PDT group at 1and 3 months (*P*=0.010; 0.009). 13/69 (18.84%) and 9/79 (11.39%) patients underwent treatment at least twice in the SMTL and half-dose PDT group, respectively, and achieved resolution of SRF after treatment. The results of subgroups analysis showed no significant differences between the logMAR BCVA of the SMTL and PDT group which were divided into parafoveal and subfoveal leakage groups after treatment, but significant difference in CRT between subgroups can be found after treatment at 1and 3 months (*P*=0.003; 0.04).

**Conclusions:**

The SMTL can be an effective candidate for the treatment of persistent cCSC where leakage occurred at parafoveal or subfoveal and improvement of logMAR BCVA, but half-dose PDT has been more effective for resolution of SRF.

## 1. Introduction

Central serous chorioretinopathy (CSC) is a relatively common chorioretinal disease characterized by serous detachment of the neurosensory retina caused by leakage from the decompensated retinal pigment epithelium (RPE) associated with mild to moderate vision loss. CSC has two distinguished manifestation forms including acute and chronic forms. Most acute CSC commonly occurs in younger patients and is thought to be self-limiting. However, the risk of recurrence for acute CSC could be about 50%, and 15% might develop persistent subretinal fluid (SRF); a duration longer than 4 months would be defined as persistent chronic CSC [1, 2]. For cCSC, subfoveal and parafovea1 leakage at the level of the RPE usually exist persistently. Unresolved SRF can lead to photoreceptor and RPE dysfunction, resulting in loss of central vision.

Different options have been suggested for treatment of CSC [3]. Although the efficacy of conventional laser photocoagulation can be observed, with a resolution of SRF for extrafoveal leakage, potential side effects including visual scotoma, reduced contrast sensitivity, and choroidal neovascularization cannot be ignored.

Furthermore, this procedure cannot be used for subfoveal or parafoveal leakage. Until now, the two most commonly used treatments for cCSC, which RPE leakage identified would be subfoveal or parafovea1, which are ICG-guided half-dose photodynamic therapy (PDT) and subthreshold micropulse treatment laser (SMTL). [2–6].

In recent years, the SMTL system is commonly used for treatment of CSC. Romano et al. concluded that both reduced-dose or reduced-fluence PDT and SMTL can reduce leakage activity in the RPE and enhance photopic and scotopic visual acuity in CSC patients [7]. The PLACE No.3 study showed that the half-dose PDT group can achieve a more favorable outcome than the high-density subthreshold micropulse laser (HSML) group using an 810 nm diode laser for the treatment of cCSC. [8].

However, in another recent study, Scholz et al. compared the efficacy and safety of SMTL and half-dose PDT in patients with cCSC and found that the STML group can acquire significantly higher morphological response 6 weeks after treatment [9]. However, no significant evidence can determine which treatment is more effective than the other until now.

In our present study, we retrospectively reviewed all cCSC patients who underwent SMTL or half-dose PDT treatment with macular subfoveal and parafoveal RPE leakage to compare the efficacy of the two different treatments for cCSC. To the best of our knowledge, the present study is one of the largest case series in one unit for evaluating and comparing the SMTL and half-dose PDT treatment for cCSC with a relatively long follow-up.

## 2. Patients and Methods

### 2.1. Study Design

This study is a retrospective, single-center, interventional longitudinal、observational study conducted on cCSC patients treated with SMTL or half-dose PDT. Approval for the study protocol was obtained from the Medical Ethics Committee of Shenzhen Eye Hospital Affiliated to Jinan University, Shenzhen Eye Disease Prevention & Treatment Institute (ethics number: 20180509), which was performed in accordance with the Declaration of Helsinki.

Written informed consent was obtained from each patient before receiving SMTL or PDT.

### 2.2. Participants

This retrospective study included patients diagnosed with cCSC and treated with SMTL or half-dose PDT between May 2014 and May 2019. For the patient with both eyes undergoing treatment, only the one with poorer visual acuity was chosen in this study. The treatment was chosen by doctors in agreement with patients. cCSC was defined based on the previous literature as follows: (1) patients showing macular serous retinal detachment on SD-OCT ≥1 area of multifocal and parafoveal or subfoveal RPE leakage on fundus fluorescein angiography (FFA) and the corresponding hyperfluorescence area on indocyanine green angiography (ICGA); (2) patients with symptomatic CSC lasting longer than 4 months [9, 10]. The patients diagnosed with other retinal diseases, such as neovascular AMD, serous-hemorrhagic PED, or polypoidal choroidal vasculopathy (PCV) were excluded.

### 2.3. Procedures

All patients underwent ophthalmologic examinations including best-corrected visual acuity (BCVA) using logMAR charts, slit-lamp microscopy, microperimetry (MP-3, NIDEK, Japan), central retinal thickness (CRT) measured by spectral-domain optical coherence tomography (SD-OCT) (Cirrus HD OCT-5000, Zeiss, Jena, Germany), fundus fluorescein angiography (FFA), fundus autofluorescein (FAF), and indocyanine green angiography (ICGA) (Spectralis HRA, Heidelberg Engineering, Heidelberg, Germany) before and after treatment. The logMAR BCVA and CRT of two groups at 1, 2, and 3 months after treatments were defined as primary outcomes. And the results of subgroups were also delivered. The secondary outcomes include the number of repetitions, and the adverse events that happened during the follow-up period were also reviewed.

**Figure 1:**
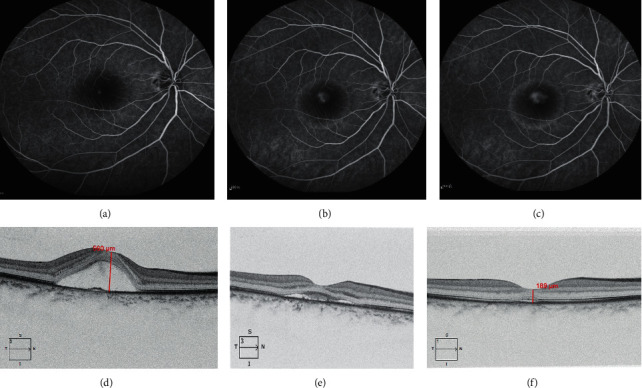
A 40-year-old male patient who underwent SMTL. (a–c) FFA showed the right eye macular subfoveal fluorescein leakage at the early phase stage, and dye formed an umbrella pattern of fluorescein pooling in the subretinal space at the late phase stage. (d) OCT showed the baseline serous macular retinal detachment combined with mini RPE detachment before SMTL. (e, f) Macular subretinal fluids partially and incompletely absorb post SMTL follow-up one and three months.

**Figure 2:**
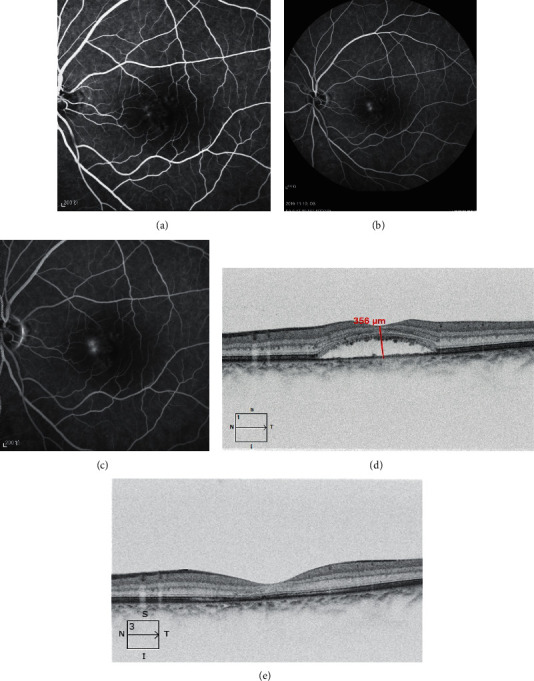
A 50-year-old male patient who underwent half-dose PDT. (a–c) FFA showed left eye macular parafoveal fluorescein leakage and RPE “windows defect” at the early phase stage, and dye formed the link pattern of fluorescein staining in the parafoveal area at the late phase stage. (d) OCT showed the baseline serous macular retinal detachment before half-dose PDT. (e) Macular subretinal fluids completely absorb after treatment follow-up three months.

The STML with the 577 nm yellow laser system (Supra Scan, Quantel Medical, Cedex, France) and half-dose PDT were both performed by a single retina specialist (QS Chen). For the protocol of the SMTL, the parameters were as follows: spot size 140–160 *µ*m and 200 ms exposure time with a 5% duty cycle (DC). The multispot model without spacing between the spots was chosen. The photocoagulation energy for each patient was titrated at the nasal upper quadrant of the retina in the monospot micropulse model. The power titration would be gradually increased until a visible burn was seen, and then, the power was reduced by 50% for individual treatment (Figure 1, 1(a)–1(f) [9–12]. For the half-dose PDT group, patients received an intravenous infusion of 3 mg/m^2^ verteporfin (Visudyne, Novartis, Basel, Switzerland) delivered over a period of 10 minutes and the treatment was ICGA-guided and performed 15 min after the start of the infusion. The PDT was applied using standard parameters (fluency, 50 J/cm^2^; wavelength, 689 nm; and exposure time, 83 seconds) (Figure 2:1(a)–1(e)) [13]. Patients with subretinal fluid (SRF) presented within the macular area were treated for both groups.

### 2.4. Statistical Analysis

Statistical analysis was performed by SPSS 24.0 (SPSS Inc., Chicago, IL, USA), and data are presented as the mean ± SD. The baseline demographics and clinical data were compared between the SMTL group versus the half-dose PDT group. Subgroups with parafoveal or subfoveal leakage also were analyzed. Repeated measures of ANOVA and the Wilcoxon signed-rank test for continuous variables were applied, and the Pearson *χ*^2^ test was used for binary variables. A *p* value of less than 0.05 was considered statistically significant.

## 3. Results

### 3.1. A Total of 148 Patients (148 Eyes) with Persistent cCSC Were Included in This Retrospective Study

Among them, 79 patients (79 eyes) were treated with half-dose PDT and 69 patients (69 eyes) were treated with the SMTL. There were 117 males (79.05%) and 31 females (20.95%) with a mean age of 44.20 ± 7.42 years (range 21–76 years). The baseline characteristics of patients in the two groups are summarized in Table 1. No significant difference in age, gender, baseline BCVA, and CRT (between the half-dose PDT group and the SMTL group) can be found.

### 3.2. LogMAR BCVA at Baseline and the Changes in Follow-Up after Treatment of Two Groups

For the SMTL group, log MAR BCVA improved from 0.46 ± 0.28 of baseline to 0.37 ± 0.26, 0.26 ± 0.20, and 0.26 ± 0.26 at 1, 2, and 3 months after treatment (*P*=0.019; *p* ≤ 0.001; *p* ≤ 0.001), while that of the half-dose PDT group improved from 0.51 ± 0.25 of baseline to 0.39 ± 0.27, 0.32 ± 0.26, and 0.26 ± 0.29 (*P*=0.024; 0.025; 0.033). No significant difference can be found between the two groups post treatment at 1, 2, and 3 months (*P*=0.723; 0.139; 0.896).

### 3.3. LogMAR BCVA Changes in Parafoveal and Subfoveal Subgroups of Two Groups

**Figure 3:**
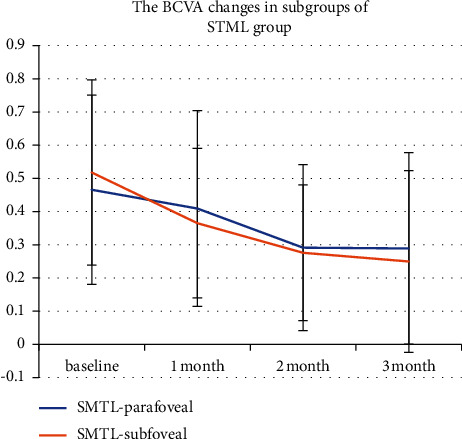
Best-corrected visual acuity (BCVA) changes in the parafoveal and subfoveal subgroups in the SMTL. The BCVA at the baseline and 1, 2, and 3 months during the follow-up period is shown in this figure. The parafoveal and subfoveal subgroups in the SMTL group analysis revealed that the treatment could improve visual outcomes. The change in BCVA in the SMTL-subfoveal group was defined with a red line chart, while the change in BCVA in the parafoveal group was defined with a blue line chart (*P*=0.582; 0.605; 0.837; 0.674).

**Figure 4:**
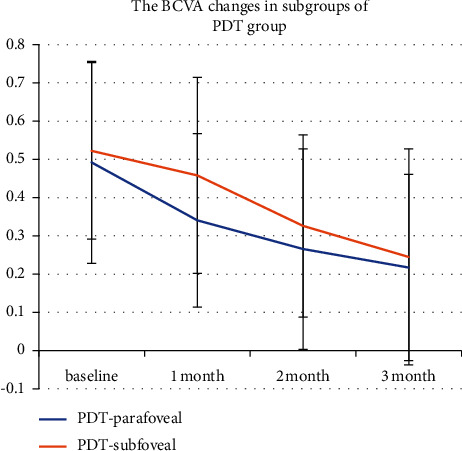
Best-corrected visual acuity (BCVA) changes in the parafoveal and subfoveal subgroups in half-dose PDT. The BCVA at the baseline and 1, 2, and 3 months during the follow-up period is shown in this figure. The parafoveal and subfoveal subgroups in the PDT group analysis revealed that the treatment could improve visual outcomes. The change in BCVA in the PDT-subfoveal group was defined with a red line chart, while the change in BCVA in the parafoveal group was defined with a blue line chart (*P*=0.648; 0.107; 0.368; 0.691).

The results of BCVA of the parafoveal and subfoveal subgroup in the SMTL group analysis revealed both treatments could improve visual outcomes, but there was no significant difference after treatment in the subgroups (*P*=0.582; 0.605; 0.837; 0.674) (Figure 3), also as same as in the half-dose PDT subgroups (*P*=0.648; 0.107; 0.368; 0.691) (Figure 4).

### 3.4. CRT at Baseline and the Changes in Follow-Up after Treatment of Two Groups

For the SMTL group, the CRT decreased from 410 ± 136 *μ*m of baseline to 347 ± 171 *μ*m, 288 ± 114 *μ*m, and 251 ± 94 *μ*m at 1, 2, and 3 months after treatment, while that of the half-dose PDT group decreased from 353 ± 133 *μ*m to 284 ± 147 *μ*m, 263 ± 90 *μ*m, and 206 ± 25.56 *μ*m. No significant difference can be found between the two groups at 2 m after treatment. However, there was a significant difference at 1 and 3 months after treatment (*P*=0.01; 0.08; 0.009), and the half-dose PDT group had a more effective decrease in CRT at the follow-up, respectively.

### 3.5. CRT Changes in the Parafoveal and Subfoveal Subgroups of Both Groups

**Figure 5:**
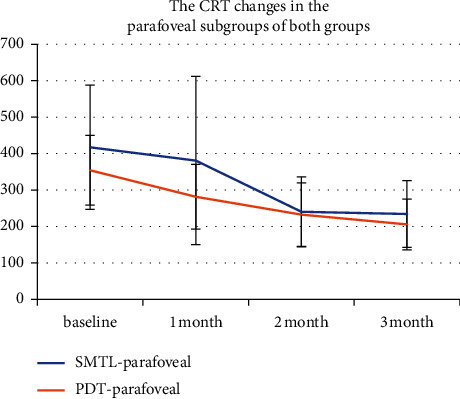
Central retinal thickness (CRT) changes in the parafoveal subgroup in the SMTL and half-dose PDT groups. The CRT at the baseline and 1, 2, and 3 months during the follow-up period is shown in the figure. The change in CRT in the PDT-parafoveal group was defined with a red line chart, while the change in CRT in the SMTL-parafoveal group was defined with a blue line chart. There was a significant difference at 1 m between the two subgroups after treatment (*P*=0.024).

**Figure 6:**
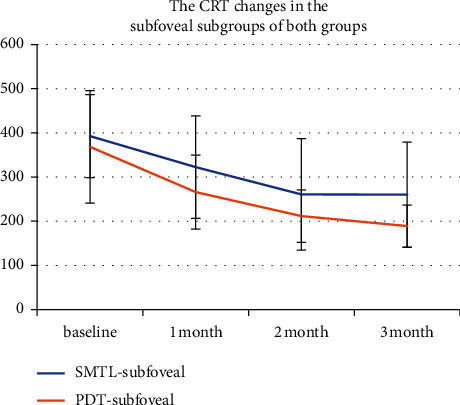
Central retinal thickness (CRT) changes in the subfoveal subgroup in the SMTL and half-dose PDT groups. The CRT at the baseline and 1, 2, and 3 months during the follow-up period is shown in the figure. The change in CRT in the PDT-subfoveal group was defined with a red line chart, while the change in CRT in the SMTL-subfoveal group was defined with a blue line chart. There was a significant difference at 1. 3 m between the two subgroups after treatment (*P*=0.03, 0.04).

The results of CRT of the parafoveal and subfoveal subgroups in the SMTL group analysis revealed that no significant difference was found at baseline or after treatment (*P*=0.432; 0.286; 0.991; 0.503), also as same as in the half-dose PDT group(*P*=0.763; 0.655; 0.453; 0.865). However, there was a significant difference at 1 m in the parafoveal subgroup between the two groups after treatment (*P*=0.024) (Figure 5). Also, a significant difference at 1, 3 m was found in the subfoveal subgroup between the two groups after treatment (*P*=0.03; 0.04) (Figure 6).

### 3.6. Re-Treatment times with Both Groups

Among the 148 cCSC patients in the study, 22 cases (14.86%) required re-treatment. For the SMTL group, 13/69 (18.84%) patients underwent treatment at least twice, while 9/79 (11.39%) patients of the half-dose PDT group received re-treatment more than once at 3 months follow-up (*P*=0.004). There was a difference between the two groups in re-treatment times.

### 3.7. Safety

No laser scars or any other structural laser damage can be observed after SMTL treatment by microperimetry or fundus autofluorescence at the end of follow-up. The laser spots can only be detected from the titration procedure in the peripheral retina. No patient in the two groups demonstrated a vision-threatening AE or systemic side effects associated with the treatments.

## 4. Discussion

As we all know, most acute CSC patients are believed to be self-limiting and can recover even without any treatment. However, for the type of chronic CSC, permanent visual loss frequently happened; therefore, PDT was more frequently used for the treatment of CSC and used in parafoveal and subfoveal lesions in the past years. For patients treated with half-dose PDT, significantly better morphologic and functional outcomes can be achieved because the PDT targets the choroidal rather than retinal tissue [13, 14]. Half-dose verteporfin PDT or low-fluence PDT has become the “gold standard” for the treatment of chronic CSC, but, on the other hand, side effects such as choriocapillaris ischemia, choroidal neovascularization, and RPE atrophy are also observed in the patients treated with PDT; moreover, PDT therapy was relatively expensive and protocol complicated. The previous study conclusion on half-dose PDT vs a high-density diode micropulse laser showed that half-dose PDT had more effective increasing visual outcomes and reducing CRT [2, 7, 8, 15]. However, clinical research by Scholz et al. revealed the 577 nm yellow laser STML had well response treatment for visual acuity and a greater decrease in CRT than half-dose PDT. The reason was PLACE studies used infrared 810 nm wavelength, leakage spot from the central fovea for 500 *μ*m, so the performed minimal titration energy could not be adequate and affected this study's outcome. Thus, the 577 nm laser is poorly absorbed by xanthophyll pigment, so we performed enough energy that could be used for treatment of the parafoveal or subfoveal area [2, 16–18].

According to our study, we evaluated the efficacy of SMTL and half-dose PDT in treatment for cCSC with parafoveal or subfoveal leakage. Both treatments showed a significant reduction of CRT and increase in BCVA than that of the baseline, and also, there were no significant differences during the following periods in two groups.

The results in our study showed mostly similar better treatment response to the SMTL and half-dose PDT different from other previous studies [7, 16] Because we only included patients with persistent SRF for at most 12 weeks and focal or multi-RPE leakage, no results for the typical chronic form of CSC were found such as irregular mildly atrophic RPE changes and choroidal abnormalities with diffuse leakage.

Some studies with a micropulse diode laser (810 nm) or 532 nm transfoveal subthreshold micropulse laser option showed some efficacy in CSC patients with extrafoveal and subfoveal leakage [3, 18, 20], and the 577 nm yellow laser STML was poorly absorbed by macular foveal xanthophyll pigments. So, it has been suggested that we induce the production of intrasellar biological factors that simulate the RPE function without causing visible damage to the retina [19]. Hence, the SMTL was more suitable option treatment for persistent cCSC with parafoveal or subfoveal leakage. In our study, we compared half-dose PDT with the SMTL to treat cCSC with parafoveal or subfoveal leakage, and the BCVA results demonstrated that both options have similar effects for subgroup persistent cCSC.

In this study, the morphological response was defined as a complete resolution of SRF 12 weeks after treatment, which was significantly higher in the half-dose PDT group. Moreover, the half-dose PDT treatment led to a significant decrease in CRT compared with SMTL after treatment at 4 and 12 weeks in subgroups, and the RPE leakage was found in the parafoveal and subfoveal group. The results were not consistent with those of the study by Breukink et al. [16].

Both groups have repeat-treated patients; for the SMTL group, 13/69 (18.84%) patients underwent treatment at least twice, while 9/79 (11.39%) patients of the half-dose PDT group received re-treatment more than once at 3-month follow-up. There was a difference between the two groups in treatment times. However, re-treated patients in the SMTL group were performed within 3 months. Once RPE leakage was incompletely resolved, SMTL was given repeatedly during the follow-up, 1 or 2 months after the first treatment, while half-dose PDT required a 3-month interval between the treatment and retreatment. This could indicate that SMTL should be initiated earlier for the best treatment outcome. This finding should be verified in a larger cohort.

There are limitations of the present study that must be mentioned. First, it is retrospectively designed and not randomized. The patients with diagnosis of cCSC treated with SMTL or half-dose PDT were all collected. Secondly, the patients included in this study have an irregular follow-up period, though the observation period of this observational study is relatively longer than that of some other studies. Thirdly, the sample size of both groups is relatively small, leading to lower strength of convincing. Undoubtedly, prospective, multicenter, larger-scale randomized controlled trials are still required to investigate and compare the efficacy of SMTL and half-dose PDT for the treatment of cCSC.

In conclusion, SMTL provides another choice for the treatment of persistent cCSC, which shows promising efficacy in our present study as well as in some previous studies. Our study can enhance the evidence of the efficacy of SMTL in cCSC patients with resolution of SRF and improvement of BCVA. Further prospective randomized studies with a larger sample are required to confirm their efficacy and safety and make further conclusions.

## 5. Conclusions

In our study, clinical outcomes of persistent cCSC patients following SMTL or half-dose PDT treatment with a follow-up period of at least 3 months demonstrate only few patients required repeat treatment, while most patients achieved significant improvement in logMAR BCVA, CRT, and resolution of SRF compared with that of the baseline.

## Figures and Tables

**Table 1:** Baseline characteristics of the SMTL and half dose-PDT groups.

	SMTL	Half-dose PDT	*P*
Number (eyes)	69	0 79	
Male, *n* (%)	57 (82.60)	0 60 (75.95)	1.000
Age (years) ,mean33 ± SD	44.33 ± 8.01	0 46.76 ± 8.43	0.200
LogMAR BCVA	0.456 ± 0.283	0 0.510 ± 0.251	0.201
CRT (*µ*m)	410 ± 136	354 ± 133	0.051

## Data Availability

All the data used to support the findings of this study are included within the article and are available from the corresponding author upon reasonable request.

## References

[1] Liu D. T., Fok A. T., Lam D. S. (2012). An update on the diagnosis and management of central serous chorioretinopathy. *Asia-Pacific Journal of Ophthalmology*.

[2] van Dijk E. H. C., Fauser S., Breukink M. B. (2018). Half-dose photodynamic therapy versus high-density subthreshold micropulse laser treatment in patients with chronic central serous chorioretinopathy. *Ophthalmology*.

[3] Sartini F., Figus M., Nardi M., CasiniPosarelli C. (2019). Non-resolving, recurrent and chronic central serous chorioretinopathy: available treatment options. *Eye*.

[4] Ambiya V., Kumar A. (2020). Role of 532 nm transfoveal subthreshold micropulse laser in non-resolving central serous chorioretinopathy with subfoveal leaks. *Therapeutic Advances in Ophthalmology*.

[5] van Rijssen T. J., van Dijk E. H., Yzer S. (2019). Central serous chorioretinopathy: towards an evidence-based treatment guideline. *Progress in Retinal and Eye Research*.

[6] Gülkaş S., Şahin O. (2019). Current therapeutic approaches to chronic central serous chorioretinopathy. *Turkish Journal of Orthodontics*.

[7] Romano M. R., Parolini B., Allegrini D. (2019). An international collaborative evaluation of central serous chorioretinopathy: different therapeutic approaches and review of literature. The European Vitreoretinal Society central serous chorioretinopathy study. *Acta Ophthalmologica*.

[8] van Rijssen T. J., van Dijk E. H. C., Scholz P. (2019). Focal and diffuse chronic central serous chorioretinopathy treated with half-dose photodynamic therapy or subthreshold micropulse laser: Place trial report No. 3. *American Journal of Ophthalmology*.

[9] Scholz P., Ersoy L., Boon C. J., Fauser S. (2015). Subthreshold micropulse laser (577 nm) treatment in chronic central serous chorioretinopathy. *Ophthalmologica*.

[10] Gemenetzi M., De Salvo G., Lotery A. J. (2010). Central serous chorioretinopathy: an update on pathogenesis and treatment. *Eye*.

[11] Zhou L., Chong V., Lai K. (2019). A pilot prospective study of 577-nm yellow subthreshold micropulse laser treatment with two different power settings for acute central serous chorioretinopathy. *Lasers in Medical Science*.

[12] Kim Y. J., Kim S. Y., Ha S. (2019). Short-duration multiple-session subthreshold micropulse yellow laser (577 nm) for chronic central serous chorioretinopathy: results at 3 years. *Eye*.

[13] Lai T. Y. Y., Chan W. M., Li H., Lai R. Y., Liu D. T., Lam D. S. (2006). Safety enhanced photodynamic therapy with half dose verteporfin for chronic central serous chorioretinopathy: a short term pilot study. *British Journal of Ophthalmology*.

[14] Lim J. I., Glassman A. R., Aiello L. P., Chakravarthy U., Flaxel C. J., Spaide R. F. (2014). Collaborative retrospective macula society study of photodynamic therapy for chronic central serous chorioretinopathy. *Ophthalmology*.

[15] Seng C.-C., Chen S.-N. (2015). Long-term efficacy of half-dose photodynamic therapy on chronic central serous chorioretinopathy. *British Journal of Ophthalmology*.

[16] Breukink B., Mohr J. K., Ossewaarde-van Norel A., den Hollander A. I., Keunen J. E., Boon C. J. (2016). Half-dose photodynamic therapy followed by diode micropulse laser therapy as treatment for chronic central serous chorioretinopathy: evaluation of a prospective treatment protocol. *Acta Ophthalmologica*.

[17] Breukink M. B., Mohr J. K., Ossewaarde-van Norel A. (2016). Half-dose photodynamic therapy followed by diode micropulse laser therapy as treatment for chronic central serous chorioretinopathy: evaluation of a prospective treatment protocol. *Acta Ophthalmologica*.

[18] Scholz P., Altay L., Fauser S. (2016). Comparison of subthreshold micropulse laser (577 nm) treatment and half-dose photodynamic therapy in patients with chronic central serous chorioretinopathy. *Eye*.

[19] Hmed A. (2015). Subthreshold micropulse yellow laser treatment for nonresolving central serous. *Chorioretinopathy Clinical Ophthalmology*.

[20] Chen C., Hwang J.-F., Hwang Li-F., TsengLin C. J. (2008). Subthreshold diode micropulse photocoagulation for the treatment of chronic central serous chorioretinopathy with juxtafoveal leakage. *Ophthalmology*.

